# The Quaternary Structure of the Recombinant Bovine Odorant-Binding Protein Is Modulated by Chemical Denaturants

**DOI:** 10.1371/journal.pone.0085169

**Published:** 2014-01-07

**Authors:** Olga V. Stepanenko, Olesya V. Stepanenko, Maria Staiano, Irina M. Kuznetsova, Konstantin K. Turoverov, Sabato D’Auria

**Affiliations:** 1 Laboratory of Structural Dynamics, Stability and Folding of Proteins, Institute of Cytology of the Russian Academy of Sciences, St. Petersburg, Russia; 2 Laboratory for Molecular Sensing, IBP-CNR, Naples, Italy; 3 St. Petersburg State Polytechnical University, St. Petersburg, Russia; University of South Florida College of Medicine, United States of America

## Abstract

A large group of odorant-binding proteins (OBPs) has attracted great scientific interest as promising building blocks in constructing optical biosensors for dangerous substances, such as toxic and explosive molecules. Native tissue-extracted bovine OBP (bOBP) has a unique dimer folding pattern that involves crossing the α-helical domain in each monomer over the other monomer’s β-barrel. In contrast, recombinant bOBP maintaining the high level of stability inherent to native tissue bOBP is produced in a stable native-like state with a decreased tendency for dimerization and is a mixture of monomers and dimers in a buffered solution. This work is focused on the study of the quaternary structure and the folding-unfolding processes of the recombinant bOBP in the absence and in the presence of guanidine hydrochloride (GdnHCl). Our results show that the recombinant bOBP native dimer is only formed at elevated GdnHCl concentrations (1.5 M). This process requires re-organizing the protein structure by progressing through the formation of an intermediate state. The bOBP dimerization process appears to be irreversible and it occurs before the protein unfolds. Though the observed structural changes for recombinant bOBP at pre-denaturing GdnHCl concentrations show a local character and the overall protein structure is maintained, such changes should be considered where the protein is used as a sensitive element in a biosensor system.

## Introduction

The problem of folding a protein molecule to a native functional structure is the hallmark of protein science. The modern protein folding theory summarized in the energy landscape model illustrates the formation of a compact, highly ordered structure of globular proteins; oligomer, amorphous aggregate or amyloid fibril formation; and a dominant role for the interaction between intrinsically disordered proteins and their partners [1], [2], [3], [4]. However, our understanding of folding for proteins with β-barrels remains limited.

A large group of odorant-binding proteins (OBPs) in the lipocalin family share a common β-barrel structure with eight β-strands (residues 9–120) linked by a turn to a short α-helical domain (residues 124–141). The α-helix is followed by the 9^th^ strand of the barrel (residues 146–148) and a C-terminal tail (residues 149–157) [5], [6]. The β-barrel in OBPs encloses a ligand binding site with an internal cavity formed by hydrophobic and aromatic amino acids as well as an external loop scaffold [7], [8]. The proteins in this group are attractive as a tool in constructing optical biosensors for dangerous substances, such as toxic and explosive molecules [9], [10], [11]. The high structural plasticity of the OBP binding site allows to optimize the interactions of the protein with ligands that differ structurally from their cognate ligands [9], [12], [13].

**Figure 1:**
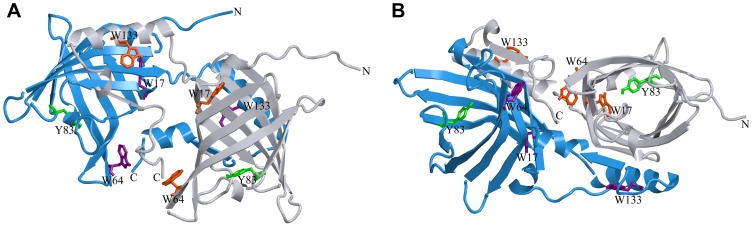
bOBP spatial pattern in two projections. The individual subunits in the protein are in gray and blue. The tryptophan residues in the different subunits are indicated in red and magenta. The conserved residue Tyr 83 is a gate for ligands [7] and is shown in green. The drawing was generated based on the 1OBP file [29] from PDB [28] using the graphic software VMD [52] and Raster3D [53].

In contrast to classic OBPs, the dimeric bovine OBP (bOBP) has an unique folding pattern that involves crossing the α-helical domain from each monomer over the β-barrel of the other monomer (Fig. 1) [6]. This dimerization/oligomerization mechanism (“domain swapping”) has been observed in many proteins and it has a great effect on protein structure and function [13], [14], [15], [16]. Domain swapping should stabilize the entire protein structure by increasing the contact area of protein matrix [14], [17], [18], [19]. In certain cases, domain swapping in proteins supports evolution of new functions that are unrelated to protein monomers [18], [20], [21], [22]. Recent data suggest that protein oligomerization through domain swapping is involved in amyloid fibril formation [15], [23], [24], [25], [26]. Thus, the bOBP dimer is a good candidate for investigating both β-barrel folding and domain swapping.

In this study, we focused our attention on the effect of guanidine hydrochloride (GdnHCl) on recombinant bOBP unfolding–refolding processes and bOBP dimerization process. The obtained results are discussed also in comparison with the stability of the native tissue bOBP.

## Materials and Methods

### Materials

GdnHCl (Nacalai Tesque, Japan), acrylamide (AppliChem, Germany), and ANS (ammonium salt of 8-anilinonaphtalene-1-sulfonic acid; Fluka, Switzerland) were used without further purification. The protein concentration was 0.1–0.2 mg/ml. The experiments were performed in 20 mM Na-phosphate-buffered solution at pH 7.8.

### Gene Expression and Protein Purification

The plasmid pT7-7-bOBP which encodes bOBP with a poly-histidine tag was used to transform *Escherichia coli* BL21(DE3) host (Invitrogen). The bOBP expression was induced by incubating the cells with 0.3 mM of isopropyl-beta-D-1-thiogalactopyranoside (IPTG; Fluka, Switzerland) for 24 h at 37°C. The recombinant protein was purified with Ni+-agarose packed in HisGraviTrap columns (GE Healthcare, Sweden). The protein purity was determined through SDS-PAGE in 15% polyacrylamide gel [27].

### Analyzing the 3D Protein Structure

We analyzed the microenvironment peculiarities for tryptophan residues in the bOBP structure based on PDB data [28] using the 1OBP PDB file [29] as described previously [30], [31].

### Fluorescence Spectroscopy

Fluorescence experiments were performed using a Cary Eclipse spectrofluorimeter (Varian, Australia) with microcells FLR (10×10 mm; Varian, Australia). Fluorescence anisotropy and lifetime were measured using a “home built” spectrofluorimeter with a steady-state and nanosecond impulse [32] as well as micro-cells (101.016-QS 5×5 mm; Hellma, Germany). Tryptophan fluorescence in the protein was excited at the long-wave absorption spectrum edge (λ_ex_ = 297 nm), wherein the tyrosine residue contribution to the bulk protein fluorescence is negligible. The fluorescence spectra position and form were characterized using the parameter 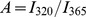, wherein 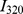 and 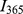 are the fluorescence intensities at the emission wavelengths 320 and 365 nm, respectively [33]. The values for parameter *A* and the fluorescence spectrum were corrected for instrument sensitivity. The tryptophan fluorescence anisotropy was calculated using the equation 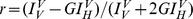, wherein 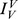 and 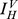 are the vertical and horizontal fluorescence intensity components upon excitement by vertically polarized light. *G* is the relationship between the fluorescence intensity vertical and horizontal components upon excitement by horizontally polarized light 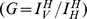, λ_em_ = 365 nm [32]. The fluorescence intensity for the fluorescent dye ANS was recorded at λ_em_ = 480 nm (λ_ex_ = 365 nm). Protein unfolding was initiated by manually mixing the protein solution (40 µl) with a buffer solution (510 µl) that included the necessary GdnHCl concentration. The GdnHCl concentration was determined by the refraction coefficient using an Abbe refractometer (LOMO, Russia; [34]). The dependences of different bOBP fluorescent characteristics on GdnHCl were recorded following protein incubation in a solution with the appropriate denaturant concentration at 4°C for 2, 24 and 48 h. bOBP refolding was initiated by diluting the pre-denatured protein (in 3.0 M GdnHCl, 40 µl) with the buffer or denaturant solutions at various concentrations (510 µl). The spectrofluorimeter was equipped with a thermostat that holds the temperature constant at 23°C. For a more detailed analysis of the protein unfolding process and to determine the number of intermediate states on the protein unfolding pathway, we used a parametric representation method for the two independent extensive parameters of the system. The parameters included denaturant concentration or time after mixing of the protein and denaturant solutions [35], [36].

### Stern-Volmer Quenching and Estimating the Bimolecular Quenching Rates

We used acrylamide-induced fluorescence quenching to evaluate the solvent accessibility of the tryptophan residues of the protein. The intrinsic protein fluorescence was excited at 297, and the emission was monitored at 340 nm. The data generated were corrected based on the solvent signal. The quenching constant was evaluated using the Stern-Volmer equation 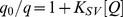, where *K*_SV_ is the Stern-Volmer quenching constant, *Q* is the quencher concentration, and the subscript 0 indicates the absence of a quencher [37], [38], [39]. Consequently, 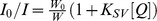, where 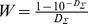, and D is the fluorophore optical density. Previous studies have demonstrated the need to include the ratio *W*_0_/*W* if the quencher absorbs at the excitation wavelength [31], [40]. The bimolecular quenching rates *k*_q_ were calculated from 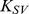 and the mean-square fluorescence lifetime τ using the equation 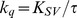 (M^−1^s^−1^) [38].

#### Circular dichroism measurements

The CD spectra were generated using a Jasco-810 spectropolarimeter (Jasco, Japan). Far-UV CD spectra were recorded in a 1-mm path length cell from 260 nm to 190 nm with a 0.1 nm step size. Near-UV CD spectra were recorded in a 10-mm path length cell from 320 nm to 250 nm with a 0.1 nm step size. For the spectra, we generated 3 scans on average. The CD spectra for the appropriate buffer solution were recorded and subtracted from the protein spectra.

#### Gel filtration experiments

We performed gel filtration experiments for bOBP in a buffered solution with addition of GdnHCl using a Superdex-75 PC 3.2/30 column (GE Healthcare, Sweden) and an AKTApurifier system (GE Healthcare, Sweden). The column was equilibrated with the buffered solution or GdnHCl at the desired concentration, and 10 µl of the protein solution prepared under the same conditions was loaded on the pre-equilibrated column. The change in hydrodynamic dimensions for bOBP was evaluated as a change in the bOBP elution volume. Multiple proteins with known molecular masses (aprotinin (6.5 kDa), ribonuclease (13.7 kDa), carbonic anhydrase (29 kDa), ovalbumin (43 kDa) and conalbumin (75 kDa), which are chromatography standards from GE Healthcare) were used to generate the calibration curve.

## Results

**Figure 2:**
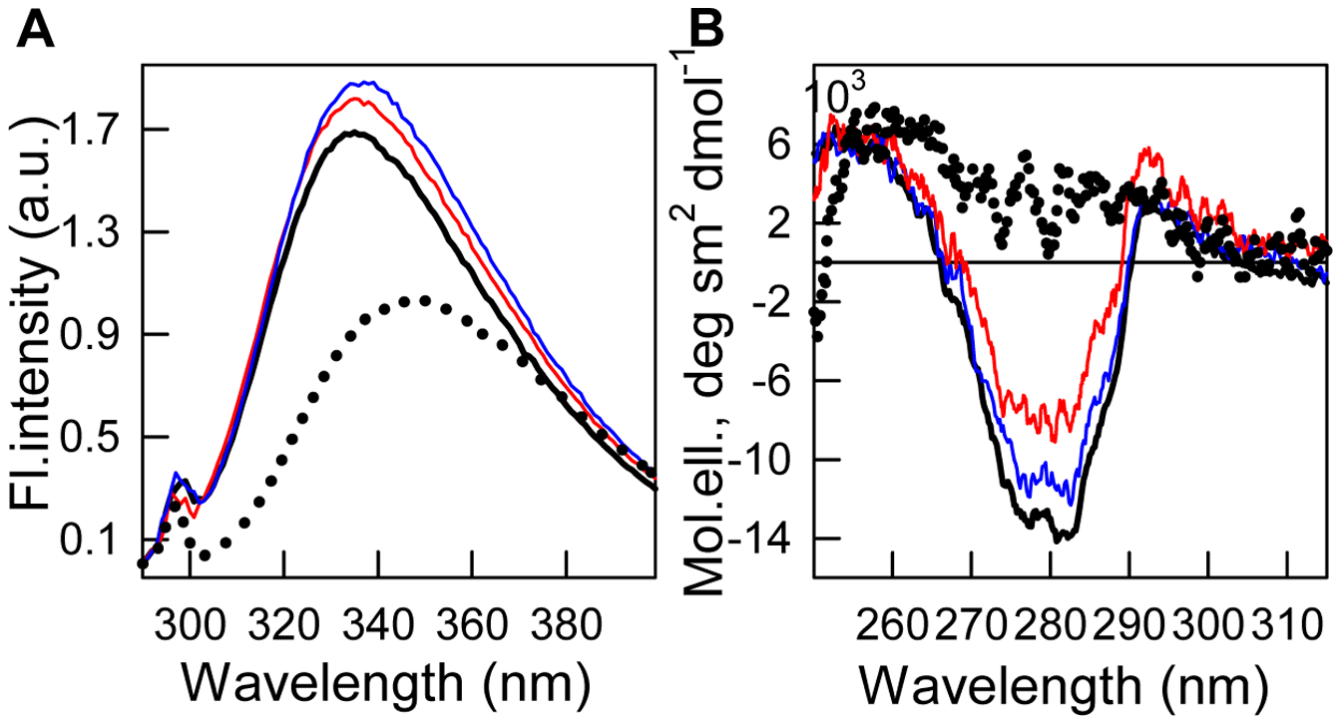
Tertiary structure changes for bOBP in different structural states are indicated by intrinsic tryptophan fluorescence (*A*, λ_ex_ = 297 nm) and near-UV CD (*B*). The spectra shown are for bOBP with GdnHCl at various concentrations: 0.0 (black solid line), 0.5 (blue solid line), 1.6 (red solid line) and 3.0 M (black dotted line).

The structural properties of the recombinant protein bOBP were investigated by using spectroscopic methods, such as intrinsic protein UV-fluorescence and far- and near-UV CD. The tryptophan fluorescence emission spectrum for bOBP is red-shifted with an emission maximum centered at 335 nm (λ_ex_ = 297 nm; Fig. 2*A*). bOBP possesses three tryptophan residues; two of them belong to the first (Trp 17) and the fourth (Trp 64) β-strands, while Trp 133 is part of the single α-helix of the protein [29].

Among all of bOBP tryptophan residues, Trp 133 presents the lowest microenvironment density (*d* = 0.54; Table S1); thus, Trp 133 is considered to be partially accessible to solvent. The microenvironment densities for the remaining tryptophan residues Trp 17 and Trp 64 are 0.80 and 0.71, respectively (Tables S3 and S5). However, Trp 17 and Trp 64 microenvironments include more polar residues if compared to Trp 133 (Tables S2, S4 and S6). It is interesting to note that the polar residues Lys 121 and Lys 59 in the Trp 17 and Trp 64 microenvironments, respectively, are parallel to the indole ring, and their amino groups NZ are closest to the indole ring NE1 atoms (5.16 and 4.55 Å for the NZ groups in Lys 121 and Lys 59, respectively, Tables S2, S4 and S6). In a previously study, we observed the presence of similar microenvironment characteristics for Trp 16 in the porcine OBP (pOBP) as well as we showed that the formation of a complex between Trp 16 and Lys 120 results in the quenching of the fluorescence of the single Trp residue present in pOBP [39], [41]. In addition, the Lys 121 and Lys 59 residues could partially quench the bOBP tryptophan fluorescence. Consequently, these microenvironment characteristics of bOBP tryptophan residues result in a red-shifted fluorescence emission spectrum [30], [33], [42], [43].

**Figure 3:**
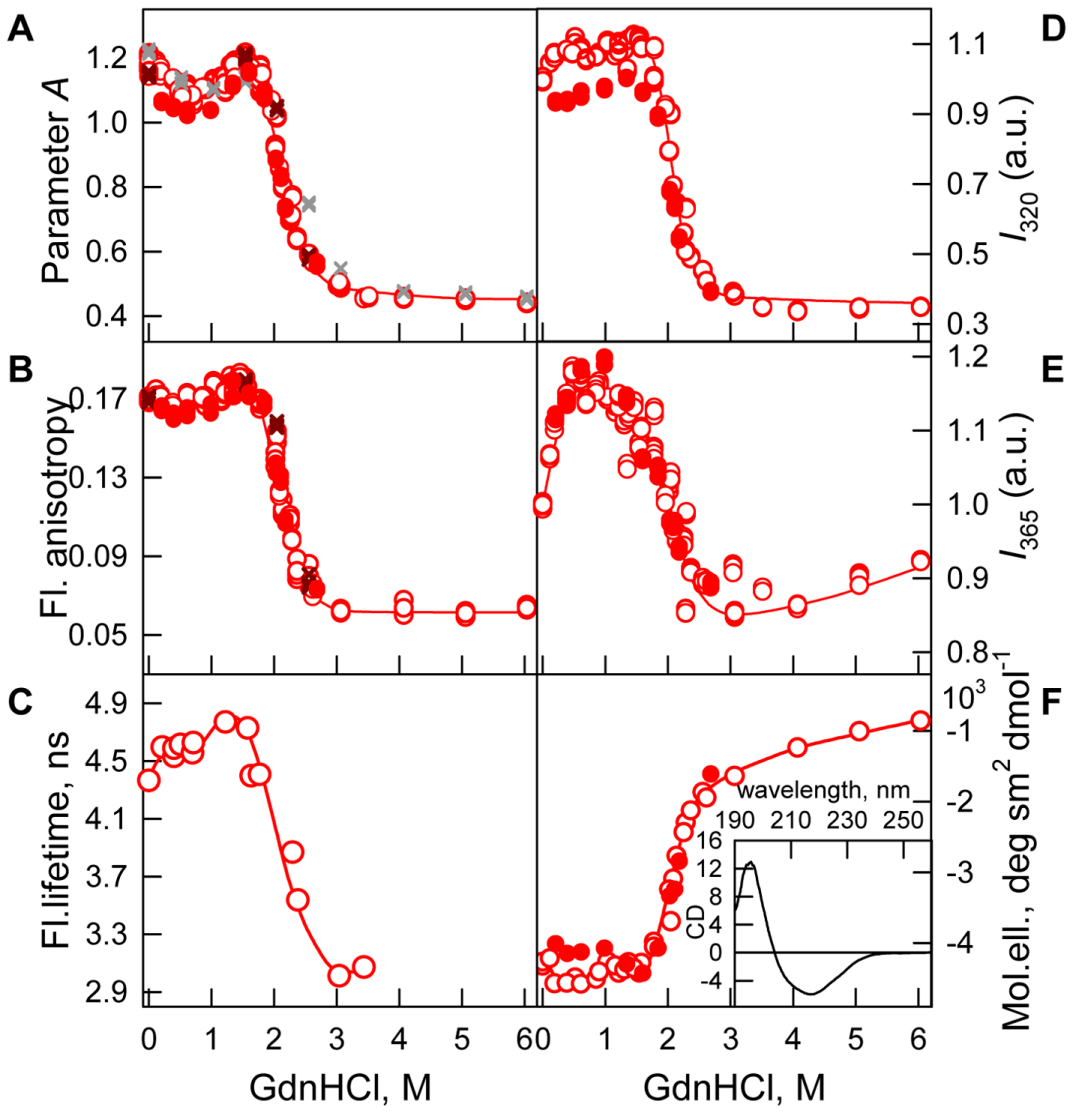
bOBP conformational changes induced by GdnHCl. ***A***: Changes in parameter *A*, λ_ex_ = 297 nm; ***B***: changes in fluorescence anisotropy at the emission wavelength 365 nm, λ_ex_ = 297 nm; ***C***: changes in the fluorescence lifetime *τ*, λ_ex_ = 297 nm and λ_em_ = 335 nm; ***D***: changes in fluorescence intensity at 320 nm, λ_ex_ = 297 nm; ***E***: changes the in fluorescence intensity at 365 nm, λ_ex_ = 297 nm; and ***F***: changes in the ellipticity at 222 nm. CD spectrum in the far-UV region for bOBP in buffered solution (insert for ***E***). The measurements were preceded by incubating the protein in a solution with the appropriate GdnHCl concentration at 4°C for 2 (gray crosses), 24 (red circles) and 48 h (brown crosses). The open symbols indicate unfolding, whereas the closed symbols represent refolding.

The protein is characterized by high fluorescence anisotropy and tryptophan fluorescence lifetime (Table 1, fig. 3, *B* and *C*). The CD spectrum in the far-UV region for bOBP has a 215 nm trough and 200 nm maximum (Fig. 3*F*, insert), which is peculiar to proteins with β-strand secondary structure. The protein also generates a marked CD spectrum in near-UV region (Fig. 2*B*), which supports a rigid and highly chiral environment for the aromatic residues, particularly the bOBP tryptophan residues in a buffered solution.

**Table 1:** Characteristics of bOBP in different structural states.

Parameter	bOBP in buffered solution	bOBP in state I_1_ (0.5 M GdnHCl)	bOBP in state I_2_ (1.6 M GdnHCl)
*Intrinsic fluorescence*			
λ_max_, nm (λ_ex_ = 297 nm)	335	337	335
Parameter *A* (λ_ex_ = 297 nm)	1.2	1.1	1.2
*r* (λ_ex_ = 297 nm, λ_em_ = 365 nm)	0.170	0.166	0.180
*τ*, nm (λ_ex_ = 297 nm, λ_em_ = 335 nm)	4.4±0.2	4.6±0.2	4.8±0.1
*Quenching by acrylamide*			
*k*_q_ at λ_em_ = 340 nm, 10^9^ M^−1^c^−1^	0.83±0.10	0.82±0.10	0.60±0.10
*K*_sv_ at λ_em_ = 340 nm, M^−1^	3.6±0.2	3.8±0.2	2.8±0.1

*GdnHCl-induced bOBP unfolding.* bOBP belongs to architectural type of proteins with β-barrel topology which are known to posses elevated resistance to different chemical denaturants [31], [39], [41], [44], [45]. In this study, we investigated the effect of GdnHCl (in the concentration range between 0.0 M and 6.0 M) on the unfolding and refolding processes of bOBP (Fig. 3).

The equilibrium dependencies for different spectral characteristics upon protein denaturation were measured after incubating bOBP for 24 h with a precisely defined concentration of GdnHCl. Prolonged incubation of the protein with the denaturing agent did not affect the bOBP spectral characteristics (Fig. 3).

The equilibrium dependences of bOBP intrinsic fluorescence and bOBP far-UV CD spectrum were measured upon the protein denaturation induced by the presence of GdnHCl. The obtained results indicate the presence of a complex shape with two clearly distinguishable two “denatured” regions in which the pattern of the different protein characteristics diverges significantly. In particular, the two “denatured” regions can be observed between 0.0 M–1.6 M GdnHCl concentration and at GdnHCl concentrations above 1.6 M (Fig. 3). For GdnHCl concentrations over 3.0 M, the protein characteristics remained unaltered and were consistent with the protein characteristics in the full unfolded state (Fig. 3). This last finding supports the bOBP structural unfolding happened in the range of concentration of GdnHCl between 1.6 and 3.0 M.

When we used pre-denaturing GdnHCl concentrations (lower than 1.6 M), we observed certain changes in bOBP intrinsic fluorescence and far-UV CD, suggesting that a complete bOBP unfolding process is headed by structural changes in the protein globule (Fig. 3).

The most marked protein structure changes at low denaturant concentrations were observed for parameter *A*, which defines the shape and position of the protein fluorescence spectrum (Fig. 3*A*). Parameter *A* decreased through 0.5 M GdnHCl, which yielded a local minimum; thereafter, parameter *A* increased with increasing the denaturant concentration, and the parameter *A* value at 1.6 M GdnHCl was characteristic of bOBP in a buffered solution. The fluorescence anisotropy for bOBP at low GdnHCl concentrations behaves similar to parameter *A* (Fig. 3*B*). However, the fluorescence anisotropy changes for bOBP under such conditions were less pronounced. As indicated in figure 3*B*, the fluorescence anisotropy for bOBP at 1.6 M GdnHCl exceeded the anisotropy for bOBP in a buffered solution (Fig. 3*B*, Table 1).

The tryptophan fluorescence lifetime for bOBP increased in a two-steps mode in the GdnHCl concentration range between 0.0 M–1.6 M (Fig. 3*C*) with a plateau at 0.2 M–0.8 M GdnHCl and a maximum at approximately 1.6 M GdnHCl for the bOBP tryptophan fluorescence lifetime curve.

Tryptophan fluorescence intensity at the bOBP emission wavelength 320 nm (*I*_320_, Fig. 3*D*) moderately increases at denaturant concentrations less than 1.6 M GdnHCl. In contrast, the tryptophan fluorescence intensity at the bOBP emission wavelength 365 nm (*I*_365_, fig. 3*E*) visibly rises with the initial denaturant increase to 0.5 M GdnHCl, which is the concentration that yields the fluorescence intensity maximum. By increasing the denaturant concentration to 1.6 M, the *I*_365_ value for bOBP decreased slightly. The *I*_365_ value for bOBP at such denaturant concentrations exceeded the value for bOBP in its initial state (Fig. 3*E*). Moderate changes in the far-UV CD for bOBP when GdnHCl concentration is less than 1.6 M indicate minor secondary structure alterations in the protein structure (Fig. 3*F*). Such changes are indicated by an initial decrease in the far-UV CD signal at 222 nm and a slight increase later.

**Figure 4:**
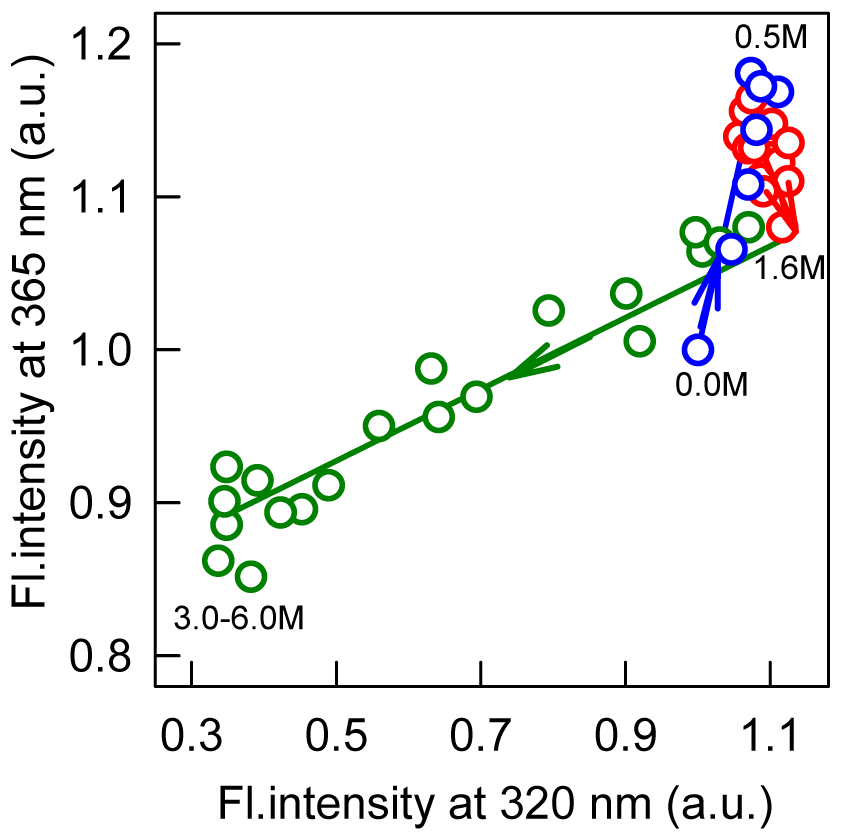
Parametric dependencies between the fluorescence intensities at 320 and 365-induced bOBP unfolding processes. The excitation wavelength is at 297–0.5 (blue), 0.5–1.6 (red) and greater than 1.6 (green).

To better understand GdnHCl-induced bOBP structural perturbation, we used a parametric representation of the two independent extensive parameters, which we have used in previous studies [35], [36], [46]. The parametric dependences of the fluorescence intensities at 320 and 365 nm for bOBP are described by a broken line composed of three individual straight lines (Fig. 4). These data suggest that GdnHCl-induced unfolding of recombinant bOBP is not a two-state process, and structural perturbations at GdnHCl concentrations below 1.6 M indicate that two intermediate states are formed at 0.5 and 1.6 M GdnHCl (Fig. 4).

Additional experiments were performed to understand the protein structure perturbations at the pre-denaturing GdnHCl concentrations. The bimolecular constants for tryptophan fluorescence quenching by acrylamide (Table 1) are low and similar for bOBP in the absence of GdnHCl or bOBP in the presence of 0.5 M GdnHCl, indicating that bOBP tryptophan residues in such states are not solvent accessible. The bimolecular constant for tryptophan fluorescence quenching by acrylamide at a concentration of GdnHCl of 1.5 M is lower (Table 1), which indicates lower solvent accessibility for the tryptophan residues in this state.

**Figure 5:**
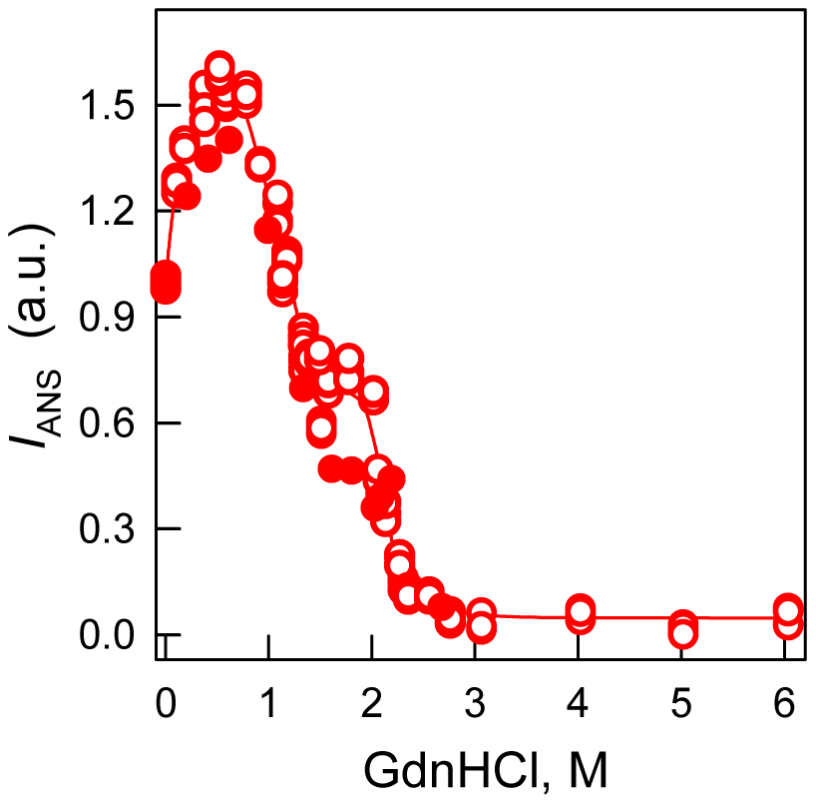
bOBP conformational changes induced by GdnHCl as indicated by the ANS fluorescence intensity. λ_ex_ = 365 nm, λ_em_ = 480 nm. The open symbols indicate unfolding, whereas the closed symbols represent refolding.

Deconvolution of the far-UV CD spectra for the recombinant protein using Provencher’s algorithm [47] showed that the secondary structural content of bOBP in the absence of GdnHCl is the following: 10% α-helices, 40% β-sheet and 21% β-turn. These values are lower than indicated by crystallographic data for wild-type bOBP (13% α-helices, 46% of β-sheets, [29]). For 0.5 M GdnHCl, the portion of secondary structure ordered elements in bOBP slightly decreased to approximately 7.0% α-helices, 38% β-sheets and 22% β-turns. With 1.5 M GdnHCl, the portion of secondary structure ordered elements in bOBP was restored to approximately 9.0% α-helices, 42% β-sheets and 25% β-turns, which is more similar to native bOBP, as indicated by the crystallographic data, than recombinant bOBP in a buffered solution [29]. For a more precise description of the bOBP intermediate state structural properties, we studied the change in intensity for the fluorescent dye ANS, which was added to a bOBP solution pre-equilibrated with GdnHCl at different concentrations (Fig. 5). The ANS fluorescence intensity for bOBP in the absence of GdnHCl was higher if compared with the dye fluorescence intensity for unfolded bOBP (3.0 M GdnHCl and above). The ANS fluorescence intensity increased with increasing the GdnHCl concentration, and the maximum emission was registered at a concentration of GdnHCl of 0.5 M. The ANS fluorescence intensity decreased at a concentration of GdnHCl of 1.4 M and reached a plateau at approximately 1.4–1.8 M GdnHCl, where the ANS intensity was lower when compared to the protein in its initial state (it is approximately 70% of the ANS fluorescence intensity for the bOBP in the absence of GdnHCl). Further increasing of the denaturant concentrations in the bOBP solution decreased the ANS fluorescence intensity to zero, which indicates that bOBP loses the ability to bind ANS during the unfolding process.

**Figure 6:**
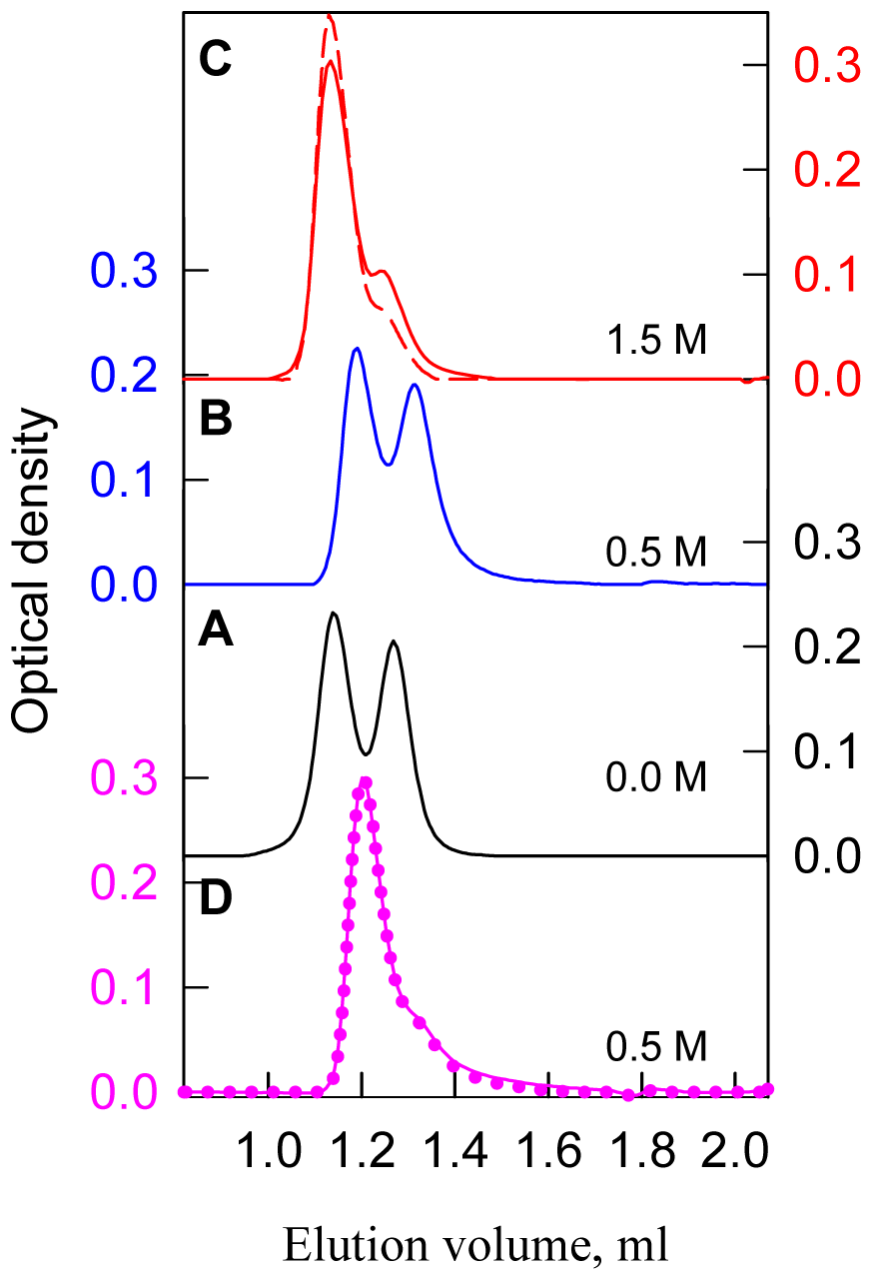
Changes in bOBP hydrodynamic dimensions for the different structural states. The elution profiles for bOBP were recorded after pre-incubation for 24 h (solid lines) and 43 h (dashed line) or 92 h (dotted line) with GdnHCl at the concentrations 0.0 (***A***, black), 0.5 (***B***, blue) and 1.5 (***C***, red) for the denaturation process and 0.5 (***D***, pink) for the refolding process after incubation with 1.5 M GdnHCl.

The bOBP structural changes for the different states were characterized by using gel filtration experiments (Fig. 6) with a Superdex75 PC 3.2/30 column. The elution profile for bOBP in the absence of GdnHCl showed two peaks with elution fractions that correspond to a protein with the molecular masses 43.9 and 23.8 kDa. These values are slightly greater than the expected values based on the protein sequence for the dimer (36.8 kDa) and monomer (18.4 kDa) protein states. At the denaturant concentration of 0.5 M GdnHCl, the bOBP elution profile includes two peaks; the peaks are clearly shifted to higher elution volumes compared with the protein in the absence of GdnHCl (Fig. 6). These data suggest that the recombinant bOBP may be represented a mixture of more compact dimers and monomers at 0.5 M GdnHCl. Through incubating bOBP in 1.5 M GdnHCl for 24 h, the protein elutes with the primary peak with the elution volume that corresponds to the first peak from the elution profile for bOBP in a buffered solution with a small shoulder at a higher elution volume. This shoulder disappears upon prolonged bOBP incubation in the denaturant solution for up to 43 h (Fig. 6); likely, under such conditions, the full recombinant bOBP population is a dimer.

Recombinant bOBP was refolded by diluting the pre-denatured protein at a high GdnHCl concentration (3.0 M) into solutions with different final denaturant concentrations. The equilibrium curves for each bOBP characteristic were observed for the protein refolding experiment and generated after incubating the protein for 24 h with GdnHCl at an appropriate concentration. These data were consistent with such characteristics for bOBP upon protein denaturation through denaturant concentrations that exceed 1.6 M (Fig. 3). At lower denaturant concentrations, we observed discrepancies in bOBP fluorescence characteristics registered after the unfolding and the refolding experiment (Fig. 3). To clarify the mechanism for such irreversibility of the protein parameters for low denaturant concentrations, we measured the elution profile after bOBP incubation in a solution with 0.5 M GdnHCl, which was generated by diluting the protein solution that was pre-incubated with 1.5 M GdnHCl (Fig. 6). The elution profile following the bOBP refolding experiment produced a single peak at the elution volume that corresponds to the first bOBP peak in 0.5 M GdnHCl from the protein denaturing process. The elution profile for the bOBP refolding experiment is not altered by prolonged incubation of the protein over several days; these data suggest that bOBP remains a dimer under the refolding experiment conditions.

## Discussion

The native bOBP extracted from native tissue is a dimer [48], [49], [50]. The dimer is constructed through a domain-swapping mechanism by crossing the α-helical domain from each monomer over the β-barrel of the other monomer [6], [50]. Such domain swapping is common for many proteins to improve protein stability and/or modulate protein function [15], [17], [18], [20], [23].

Our data obtained by gel filtration experiments, show that recombinant bOBP is a mixture of monomers and dimers under native conditions (in buffered solution), and the full recombinant bOBP population is a dimer in the presence of 1.5 M GdnHCl (Fig. 6). The bOBP elution volume under such conditions corresponds to a protein with the molecular mass 43.9 kDa; this molecular weight is slightly greater than expected for the native bOBP based on its amino acid sequence, but it is consistent with the molecular weight previously determined for the native (tissue-extracted) protein using gel filtration [48], [50]. The dimeric bOBP in the presence of 1.5 M GdnHCl has a highly ordered secondary structure, which was determined through deconvolution of far-UV CD data. The pronounced near-UV CD spectrum (Fig. 2*B*) and low bimolecular constants for tryptophan fluorescence quenching using acrylamide (Table 1) for bOBP in the presence of 1.5 M GdnHCl suggest a highly rigid microenvironment around the tryptophan residues in the dimer state. In the absence of GdnHCl, the recombinant bOBP is in a stable state with features similar to the native dimeric bOBP. Such similarity was determined through protein characteristics, such as fluorescence spectrum position, parameter *A*, and fluorescence anisotropy, for recombinant bOBP in the absence of GdnHCl and dimeric bOBP in the presence of 1.5 M GdnHCl (Table 1). In this state, the tryptophan residues are slightly more solvent accessible, which is consistent with the shorter fluorescence lifetime (Table 1) and lower fluorescence intensity (Fig. 2*A*). Additionally, more pronounced near-UV CD data (Fig. 2*B*) suggest that the tryptophan residues in the recombinant bOBP have a more rigid microenvironment in a buffered solution. A more rigid microenvironment can decrease the distance between the tryptophan residue indole rings and quench groups in their microenvironment (Tables S1–S6), which decreases the fluorescence lifetime and intensity. Recombinant bOBP in the absence of GdnHCl is characterized by a less ordered secondary structure compared with the wild-type bOBP crystallographic data. Such local alterations in the recombinant bOBP secondary and tertiary structure in a buffered solution are accompanied by a decreased capacity for recombinant bOBP dimerization in a buffered solution, which is a mixture of monomers and dimers (Fig. 6). We suppose that this stable recombinant bOBP state in buffered solution with a decreased tendency for dimerization is a “trap” state with an energy minimum that is similar to the bOBP native state and characterized by incorrect α-helical and β-sheet packing for the protein globule, which may interfere with forming the bOBP native state. Clearly, the domain-swapping mechanism that produces the native, dimeric bOBP is relatively complex and requires that the monomers are correctly folded. Our conclusion is confirmed because the native dimer state was formed in the presence of 1.5 M GdnHCl after progressing through an intermediate state, which reorganized the bOBP structure. The recombinant bOBP intermediate state was populated in the presence of 0.5 M GdnHCl. In this state, the protein has fewer ordered secondary structure elements, both α-helices and β-sheets, compared with recombinant bOBP both in a buffered solution and in solution containing 1.5 M GdnHCl. The intermediate state for recombinant bOBP in the presence of 0.5 M GdnHCl was also characterized by an ordered tertiary structure, which was confirmed by pronounced near-UV CD absorption bands (Fig. 2*B*). Increasing the denaturant concentration to 0.5 M did not change the oligomeric status for bOBP, but the protein became more compact both in its monomer and dimer forms (Fig. 6). Interestingly, previous observations showed a similar state for bOBP with ordered secondary and tertiary structures and the ability to bind ANS at acidic pH values [51]. The capacity for recombinant bOBP to bind the fluorescent dye ANS in this intermediate state (Fig. 5) is likely related to a more relaxed secondary structure. We observed some protein structure reorganization, both in the α-helical and β-sheet regions, which may facilitate further transition of the protein to the native dimer. A complete unfolding of the structure of the recombinant protein is induced by an increased denaturant concentration greater than 1.5 M GdnHCl. The high resistance to denaturation observed for the recombinant protein with an unfolding midpoint above 2 M GdnHCl is comparable to the stability for the native (tissue extracted) bOBP [49] and it is typical for β-barrel proteins [44]. Recombinant bOBP unfolding is reversible, which was indicated by similar parameters in the transition region, which were measured as unfolding and refolding progressed. However, the bOBP dimerization process that precedes protein unfolding is irreversible. The domain swapping mechanism underlying dimer formation likely has a stabilizing effect on the protein globule and maintains the correct folding pattern for dimeric bOBP under pre-denaturing concentrations. Our investigations on the recombinant bOBP stability and unfolding processes aid in discerning the fundamental folding problem for proteins with β-barrel topology. Because the dimerization process for bOBP is irreversible and requires the reorganizing of the protein structure, the protein oligomerization through the domain-swapping mechanism is complex. The changes in the recombinant bOBP structure at pre-denaturing GdnHCl concentrations have a local character with the overall protein structure conserved. These changes should be considered in case the protein is intended for use as a sensitive element in a biosensor system.

## Supporting Information

Table S1 **Side chain conformation of Trp 133 in bOBP.**
(DOC)

Table S2 **Characteristics of the Trp 133 microenvironment in bOBP.**
(DOC)

Table S3 **Side chain conformation of Trp 17 in bOBP.**
(DOC)

Table S4 **Characteristics of the Trp 17 microenvironment in bOBP.**
(DOC)

Table S5 **Side chain conformation of Trp 64 in bOBP.**
(DOC)

Table S6 **Characteristics of the Trp 64 microenvironment in bOBP.**
(DOC)
