# Supplementary material for: The Quaternary Structure of the Recombinant Bovine Odorant-Binding Protein Is Modulated by Chemical Denaturants
Source: PLoS One. 2014 Jan 7;9(1):e85169. doi: 10.1371/journal.pone.0085169 (PMC3883677; doi:10.1371/journal.pone.0085169)
Supplement: Table S1 — Side chain conformation of Trp 133 in bOBP. (DOC) [file pone.0085169.s001.doc]

**Table S1.** Side chain conformation of Trp 133 in bOBP.

| N (*d*)*** | *χ*_1_, (deg)* | *χ*_2_, (deg)* |
| --- | --- | --- |
| 56 (0.54) | 287.95 | 112.22 |

* N is the number of atoms in the microenvironment of tryptophan residue; *d* is the density of tryptophan residue microenvironment; *χ*_1_ and *χ*_2_ are the angles characterizing the conformation of tryptophan residue side chain.
