# Supplementary material for: The Quaternary Structure of the Recombinant Bovine Odorant-Binding Protein Is Modulated by Chemical Denaturants
Source: PLoS One. 2014 Jan 7;9(1):e85169. doi: 10.1371/journal.pone.0085169 (PMC3883677; doi:10.1371/journal.pone.0085169)
Supplement: Table S2 — Characteristics of the Trp 133 microenvironment in bOBP. (DOC) [file pone.0085169.s002.doc]

**Table S2**. Characteristics of the Trp 133 microenvironment in bOBP.

| residue | atom | R*, Å |
| --- | --- | --- |
| *polar groups* | | |
| Tyr 21B | OH(CZ2) | 4.36 |
| Thr 136 | OG1 (CA) | 4.35 |
| Lys 143 | NZ (CE3) | 3.35 |
| HOH 218A | (CE3/CZ3) | 4.69 |
| HOH 254A | (NE1) | 3.94 |
| HOH 232B | (NE1) | 3.27 |
| HOH 283B | (CZ2) | 5.83 |
| *peptide bonds* | | |
| Leu 129 | N(N) | 6.23 |
| Leu 129 | O(N) | 3.10 |
| Glu 130 | N(CD1) | 4.75 |
| Glu 130 | O(N) | 3.33 |
| Phe 132 | N(N) | 2.85 |
| Phe 132 | O(N) | 2.26 |
| Lys 134 | N(C) | 1.33 |
| Lys 134 | O(O) | 3.28 |
| Thr 136 | N(O) | 3.37 |
| Thr 136 | O(O) | 5.01 |
| Lys 143 | N(CZ3) | 5.56 |
| Lys 143 | O(CH2) | 5.36 |
| Val 146 | N(CH2) | 6.50 |
| Val 146 | O(CH2) | 6.63 |
| *nonpolar groups and aromatic residues* | | |
| Tyr 21B | CB, CG, CD1, CD2, CE1, **CE2**, CZ | 4.17 |
| Phe 132 | CA, **C,** CB, CG, CD1, CD2, CE1, CE2, CZ | 1.34 |
| Leu 129 | CA, **C**, CB, CG, CD1, CD2 | 3.90 |
| Lys 134 | **CA**, C, CB, CG, CD | 2.43 |
|  |  |  |
| Val 146 | CB, CG1, **CG2** | 3.69 |
| Lys 143 | CA, C, CB, **CG**, CD, CE | 3.75 |

*R is the minimal distance between a residue involved in the microenvironment of tryptophan residue and its indole ring.
