# Supplementary material for: The Quaternary Structure of the Recombinant Bovine Odorant-Binding Protein Is Modulated by Chemical Denaturants
Source: PLoS One. 2014 Jan 7;9(1):e85169. doi: 10.1371/journal.pone.0085169 (PMC3883677; doi:10.1371/journal.pone.0085169)
Supplement: Table S4 — Characteristics of the Trp 17 microenvironment in bOBP. (DOC) [file pone.0085169.s004.doc]

**Table S4**. Characteristics of the Trp 17 microenvironment in bOBP.

| residue | atom | R*, Å |
| --- | --- | --- |
| *polar groups* | | |
| Ser 14 | OG(NE1) | 6.86 |
| Arg 18 | NE(O) | 4.16 |
| Arg 18 | NH1(O) | 2.95 |
| Arg 18 | NH2(O) | 5.18 |
| Glu 42 | OE1(N) | 5.44 |
| Glu 42 | OE2(N) | 6.15 |
| Ser 95 | OG(CH2) | 6.67 |
| His 98 | ND1(CZ3) | 6.87 |
| Lys 121 | NZ (NE1) | 5.16 |
| HOH 318A | (CZ3) | 6.87 |
| *peptide bonds* | | |
| Leu 13 | O(NE1) | 2.87 |
| Ser 14 | O(NE1) | 5.05 |
| Ser 14 | N(NE1) | 4.86 |
| Gly 15 | O(N) | 3.66 |
| Gly 15 | N(NE1) | 4.43 |
| Pro 16 | N(N) | 3.54 |
| Pro 16 | O(N) | 2.25 |
| Arg 18 | N(C) | 1.32 |
| Arg 18 | O(C) | 3.95 |
| Phe 40 | O(O) | 3.26 |
| Phe 40 | N(O) | 5.10 |
| Glu 42 | N(O) | 4.22 |
| Glu 42 | O(CD1) | 5.73 |
| Leu 43 | N(CD1) | 4.02 |
| Leu 43 | O(CD1) | 4.49 |
| Ser 95 | O(CH2) | 4.14 |
| Ser 95 | N(CH2) | 5.77 |
| Arg 96 | N(CH2) | 4.71 |
| Arg 96 | O(CH2) | 6.11 |
| Thr 97 | N(CH2) | 5.09 |
| Thr 97 | O(CZ3) | 3.76 |
| His 98 | O(CH2) | 4.10 |
| His 98 | N(CZ3) | 4.14 |
| Leu 99 | N(CZ3) | 3.74 |
| Leu 99 | O(CZ3) | 5.57 |
| Phe 119 | O(CZ3) | 3.85 |
| Phe 119 | N(CE3) | 5.75 |
| Val 120 | O(CA) | 3.38 |
| Val 120 | N(CE3) | 3.92 |
| Lys 121 | O(CA) | 6.07 |
| Lys 121 | N(CE3) | 3.51 |
| Leu 122 | N(CA) | 4.61 |
| Leu 122 | O(O) | 5.97 |
| *nonpolar groups and aromatic residues* | | |
| Pro 16 | **CB**, CG, CD | 3.49 |
| Phe 40 | **CB**, CG, CD1, CD2,CE2 | 3.74 |
| Phe 45 | CG, CD1, **CE1**, CE2, CZ | 4.61 |
| His 98 | **CB**, CG | 5.32 |
| Phe 119 | **CB**, CG, CD1, CD2 | 4.13 |
| Leu 13 | CB, CG, CD1, **CD2** | 4.17 |
| Leu 43 | **CB**, CG, CD1, CD2 | 3.65 |
| Leu 94 | CB, CG, **CD1**, CD2 | 4.26 |
| Leu 99 | **CB**, CG, CD1, CD2 | 3.80 |
| Val 120 | **CB**, CG1, CG2 | 5.51 |
| Lys 121 | **CB**, CG, CD, CE | 3.83 |
| Leu 122 | CB**,** CG, **CD1**, CD2 | 4.69 |

*R is the minimal distance between a residue involved in the microenvironment of tryptophan residue and its indole ring.
