# Supplementary material for: The Quaternary Structure of the Recombinant Bovine Odorant-Binding Protein Is Modulated by Chemical Denaturants
Source: PLoS One. 2014 Jan 7;9(1):e85169. doi: 10.1371/journal.pone.0085169 (PMC3883677; doi:10.1371/journal.pone.0085169)
Supplement: Table S6 — Characteristics of the Trp 64 microenvironment in bOBP. (DOC) [file pone.0085169.s006.doc]

**Table S6**. Characteristics of the Trp 64 microenvironment in bOBP.

| residue | atom | R*, Å |
| --- | --- | --- |
| *polar groups* | | |
| Tyr 39 | OH(CH2) | 5.35 |
| Ser 57 | OG(CE3) | 5.05 |
| Lys 59 | NZ(NE1) | 4.55 |
| Lys 63 | NZ(N) | 6.58 |
| Lys 65 | NZ(O) | 5.23 |
| His 157 | ND1(CD1) | 3.27 |
| His 157 | NE2(CD1) | 4.22 |
| Glu 159 | OE1(CH2) | 5.16 |
| Glu 159 | OE2(CH2) | 4.56 |
| HOH 205A | (CH2) | 4.04 |
| HOH 231A | (CZ3) | 5.70 |
| HOH 247A | (CH2) | 5.80 |
| HOH 282A | (CD2) | 3.74 |
| HOH 289A | (CB) | 4.18 |
| HOH 298A | (CZ3) | 3.56 |
| HOH 328A | (NE1) | 2.88 |
| *peptide bonds* | | |
| Tyr 39 | N(CZ3) | 6.04 |
| Tyr 39 | O(CZ3) | 6.78 |
| Ser 57 | N(CZ3) | 5.96 |
| Ser 57 | O(CZ3) | 4.20 |
| Val 58 | N(CE3/CZ3) | 4.00 |
| Val 58 | O(C) | 3.67 |
| Lys 59 | N(CE3/CZ3) | 3.78 |
| Lys 59 | O(CA) | 6.52 |
| Arg 60 | N(N) | 5.11 |
| Arg 60 | O(N) | 5.63 |
| Lys 63 | N(N) | 3.61 |
| Lys 63 | O(N) | 2.25 |
| Lys 65 | N(C) | 1.32 |
| Lys 65 | O(C) | 4.07 |
| Pro 156 | O(CE2) | 6.63 |
| His 157 | N(NE1) | 5.98 |
| His 157 | O(CZ2) | 5.90 |
| Pro 158 | N(CZ2) | 4.47 |
| Pro 158 | O(CZ2) | 6.07 |
| Glu 159 | N(CZ2) | 4.12 |
| Glu 159 | O(NE1) | 3.31 |
|  | *nonpolar groups and aromatic residues* | |
| Tyr 39 | CB, CG, CD1, CD2, **CE1**, CE2, CZ | 3.88 |
| His 155 | CE1 | 6.40 |
| His 157 | CB, CG, CD2, **CE1** | 3.52 |
| Pro 158 | CB, CG, **CD** | 3.69 |
| Val 58 | **CB**, CG1, CG2 | 5.50 |
| Lys 63 | **CB**, CG, CD, CE | 3.23 |
| Lys 65 | **CB**, CG, CD, CE | 3.46 |

*R is the minimal distance between a residue involved in the microenvironment of tryptophan residue and its indole ring.
